# Application of PRI-E–a combined learning method in oral and maxillofacial oncology education

**DOI:** 10.1038/s41598-024-58878-y

**Published:** 2024-04-07

**Authors:** Zi-Zhan Li, Hao Lin, Yuan-Ming Xu, Qi-Wen Man, Tian-Fu Wu, Zhe Shao, Shanshan Liang, Lin-Lin Bu, Bing Liu

**Affiliations:** 1 State Key Laboratory of Oral & Maxillofacial Reconstruction and Regeneration, Key Laboratory of Oral Biomedicine Ministry of Education, Hubei Key Laboratory of Stomatology, School & Hospital of Stomatology, Wuhan University, Wuhan, China; 2 Division of Oral Medicine and Dentistry, Brigham and Women’s Hospital, Harvard School of Dental Medicine, Boston, USA; 3 Department of Oral and Maxillofacial Head Neck Oncology, School and Hospital of Stomatology, Wuhan University, Wuhan, China; 4 Department of Prosthodontics, School and Hospital of Stomatology, Wuhan University, Wuhan, China

**Keywords:** Combined learning method, Step-by-step approach, SWOT analysis, Problem-based learning, Case-based learning, Evidence-based learning, Cancer, Health care, Medical research, Oncology

## Abstract

The traditional lecture-based learning (LBL) method is facing great challenges due to its low efficiency and single proceeding form. We designed a PRI-E learning mode that combined and modified problem-based, case-based, and evidence-based learning with a step-by-step approach. We evaluated the practical learning outcomes of using the PRI-E mode by comparing it with traditional lecture-based learning in oral and maxillofacial oncology education. “PRI-E” consists of the first letters of the English words Passion, Research, Innovation, and Education, and it means “the best Education”. This prospective randomized controlled trial included 40 participants. We evenly divided the participants into the PRI-E (n = 20) and LBL group (n = 20) based on the entrance test scores. The same staff group designed and then taught the learning content with different group measures. The evaluation included the final test scores and questionnaire assessments. Without affecting the examination results, the PRI-E teaching method was more satisfactory and popular with participants in terms of ability development and classroom participation. Enacting the PRI-E teaching method required more time, but this did not affect its popularity among the participants. Compared with the LBL learning mode, the PRI-E learning mode was more organized and efficient in oral and maxillofacial oncology education without affecting academic performance. This model has a high degree of satisfaction, which is conducive to training students' comprehensive ability.

## Introduction

The current stomatological education model in China is different from that in other countries. Traditional exam-oriented lecture-based learning (LBL) is still the mainstream method of the current stomatological education model in China. The conflict between the inadequacy of LBL teaching methods and the requirements of China's medical reform is increasingly fierce. Traditional LBL requires students to listen to the teacher all times during class, which is not efficient and prevents the active exploration of knowledge^1^. Communications are inevitable in clinical scenarios. The ability to communicate and solve problems is also imperative in the training of doctors^2–4^. However, the current medical education lacks the cultivation of communication ability and problem-solving ability, which will affect the future career development of students^4^. Thus, more efficient and comprehensive learning methods need to be explored.

**Figure 1:**
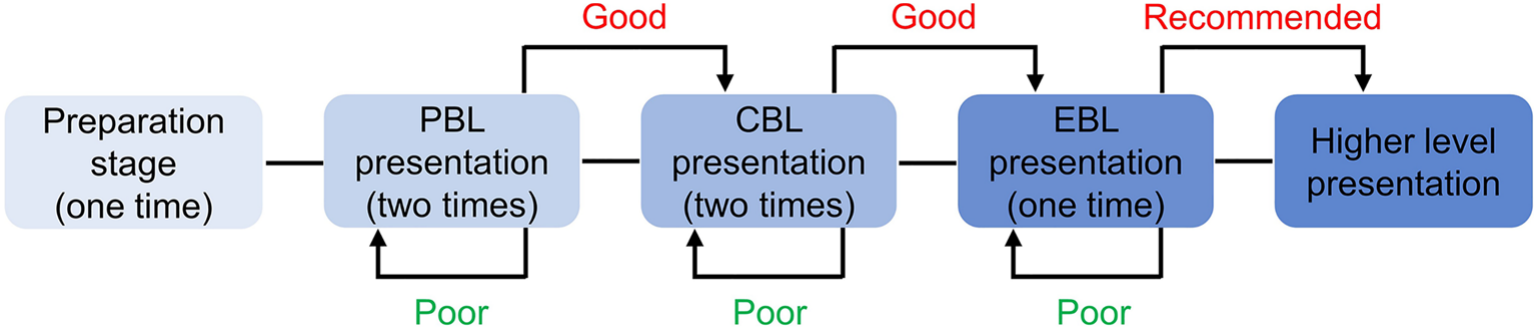
Step-by-Step flow chart. Firstly, participants start with the PBL presentation assigned by faculty. Then, participants can give CBL presentation when PBL contents were well delivered. And participants receive EBL task and take part in higher level contest.

Problem-based learning (PBL) originated in the 1960s in the medical school at McMaster University in Canada. PBL helps students construct new concepts and knowledge by solving clinical problems. It has been recognized as one of today’s most advanced learning methods and has been widely accepted^5–7^. Case-based learning (CBL) originated from Harvard University and is a way to guide students to explore and research through real-world clinical cases^1,8^. Evidence-based learning (EBL) is a learning method used to realize a perfect combination of clinical practice and scientific evidence to summarize the existing experience and guide clinical decision making to the maximum^9,10^. However, differences in their implementation exist. PBL does not require clinical experience, whereas the implementation of CBL requires students to have some clinical basic knowledge^11,12^. PBL is problem-guided learning, whereas CBL requires students to recall previous knowledge to solve the clinical cases and problems derived from them^13^. A single learning mode is compared to the traditional LBL mode in most of the literature^1,14–16^. Few scholars adopt the combination of multiple learning methods and lack the guidance of one concrete educational philosophy^17^. Thus, we modified the existing learning modes and designed a PRI-E learning method. "PRI-E" consists of the first letters of the English words Passion, Research, Innovation, and Education, and it means "the best Education". It represents a special, targeted, and systematic method that combines all the above learning methods with a step-by-step approach in oral and maxillofacial oncology teaching (Fig. 1).

*Step 1*: The participants start with the PBL presentation assigned by the faculty.

*Step 2*: The participants can give a CBL presentation when the PBL content is delivered well.

*Step 3*: The participants receive an EBL task and participate in a higher-level contest.

Passion, Research, and Innovation were the key points that we summarized from PBL, CBL, and EBL. Therefore, compared with these teaching methods, the PRI-E learning method has the following advantages and innovations: Passion can lead to deeper thinking and executive force, which is the premise of any learning activity. Research represents the desire for knowledge and highlights the rigor of learning activities. Innovation is applied to both activity forms and content, which is fundamental to PRI-E’s future development. “PRI-E” indicates that we are committed to providing the best education and lifelong learning capacity to participants, and this educational philosophy is emphasized during teaching activities. We conducted a study assessing the PRI-E method and traditional LBL method in the Wuhan University from September 2020 to March 2021. We aim to propose a new teaching method and compare it with the existing LBL and PBL learning methods. We have verified the potential of PRI-E learning method in the teaching of oral and maxillofacial oncology, and put forward the existing problems and improvement measures.

## Methods

### Participants

A total of forty students and ten teaching staff were enrolled in this study from September 2020 to March 2021 in the Department of Oral and Maxillofacial Head and Neck Oncology of School and Hospital of Stomatology, Wuhan University. 19 male and 21 female students participated in the study. All the students had finished undergraduate study of the relevant content. After receiving the scores of the entrance examination, participants will be stratified according to the score range (0–60; 61–80; 81–100), and participants in each layer will be evenly divided into PRI-E and LBL groups. The ten staff members received pre-job training and were responsible for the two groups’ teaching work.

### Study design

**Figure 2:**
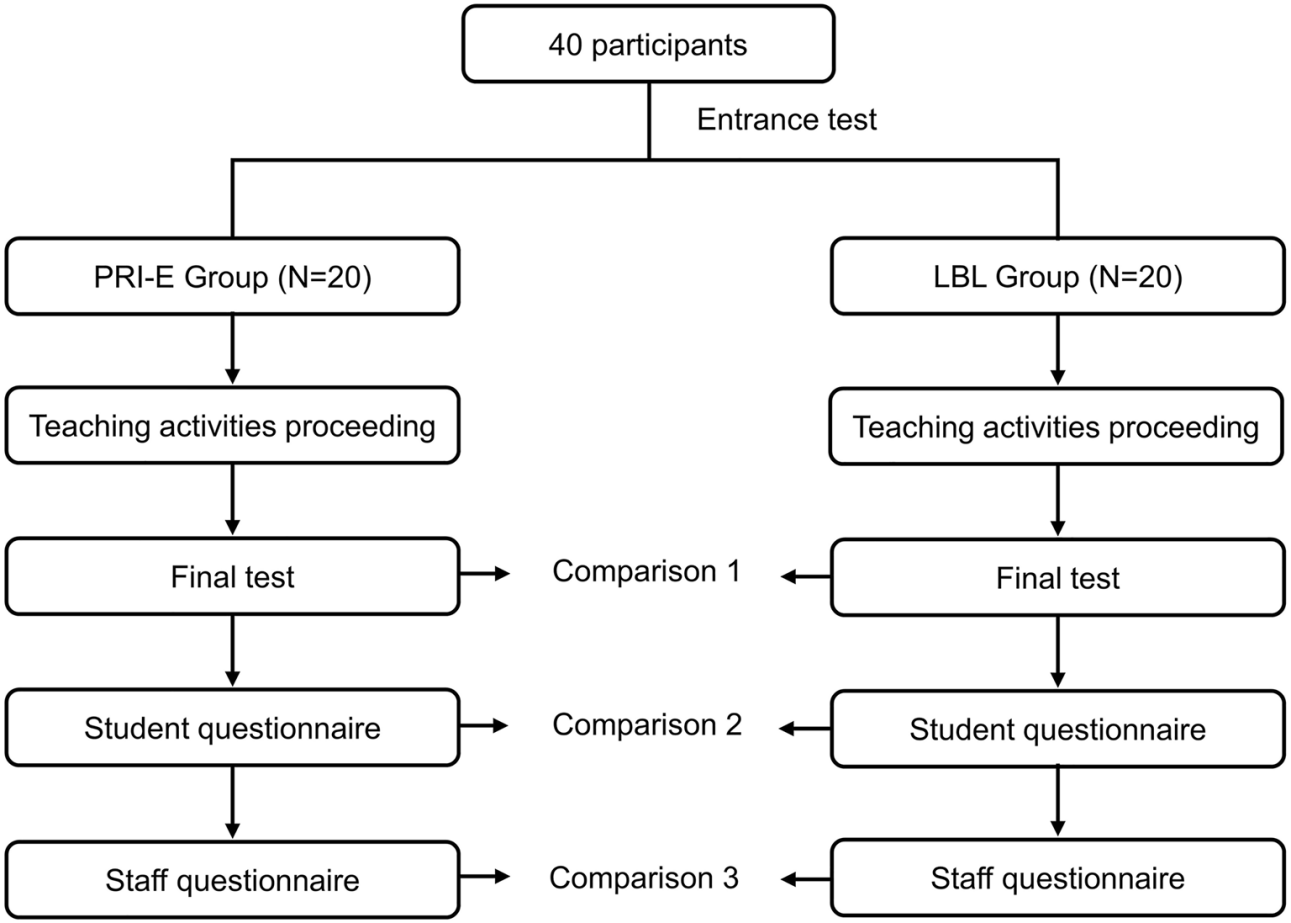
Study design flow chart. The students who participated in this study were first randomly divided into PRI-E group and LBL group in equal quantity. They went through the same four steps, including teaching acidity proceeding, final test, student questionnarie and staff questionnarie. The last three steps were compared between the two groups.

The students wrote an entrance test (Table S1) and were then evenly divided into the PRI-E and LBL group according to their test results. During the rotation, the two groups received the same learning content designed by one staff group. The learning content in the Department of Oral and Maxillofacial Head and Neck Oncology included theoretical learning and practical skill training. All the students studied for 6 months. Each learning process included three stages: before, during, and after class. At the end of the course, the students wrote a final exam (Table S2) and filled out an anonymous questionnaire (Fig. 2). The judges of the examination papers were all experienced clinicians. All the learning content and hardware equipment were well controlled to ensure an effective evaluation between the learning methods. In addition, researchers are not among the ranks of staff.

### PRI-E method

Learning contents included presentation and clinical practice.

#### Presentation

**Figure 3:**
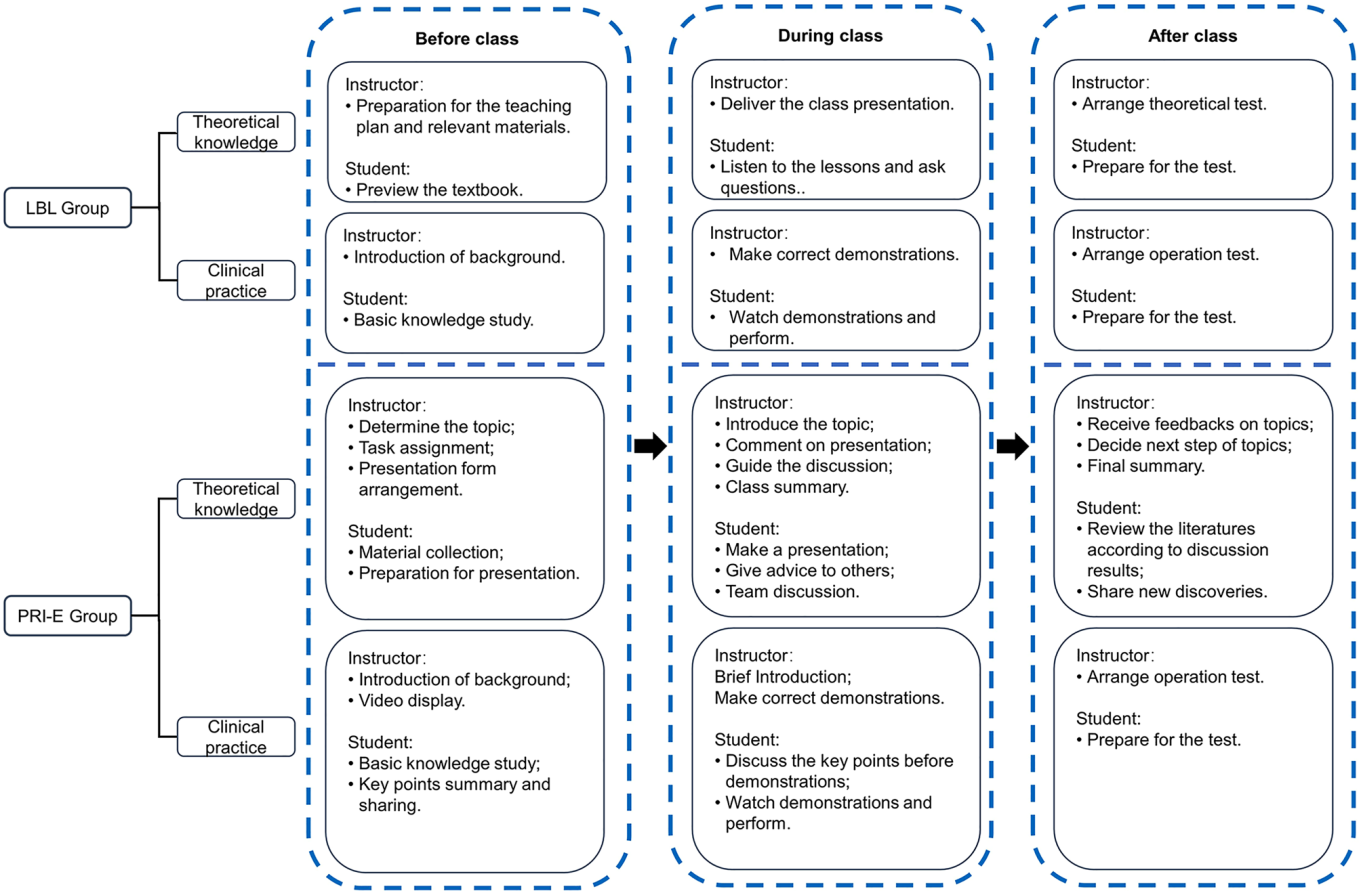
Detailed learning procedure. Both PRI-E group and LBL group needed theoretical knowledge and clinical practice. The learning of the two groups went through three stages: before class, during class and after class. The duration of each of the two teaching methods is consistent (80 min), including before (10 min), during (60 min), and after (10 min). During the six-month rotation of learning, the frequency of the two learning methods is once a week.

The staff introduced the plan and learning content in the first class. The presentation topics were based on the syllabus and clinical experience. The presentation process followed a step-by-step approach that was designed according to the real experiences of each student (Fig. 1). PBL required little clinical experience and focused on every small clinical issue, which may have been helpful for the students to build confidence. The students were not assigned a CBL or EBL presentation at the beginning as they both required a heavier workload and more experience. After the students delivered the presentation, the other students or staff members were asked to offer advice regarding the content and fluency of the presentation. The participants then engaged in a discussion about the content. If any controversy or new discovery was present, the presentation form could be repeated for further exploration and discussion. Students were also allowed to repeat one presentation form if the speech was not satisfying, which prevented some participants from finishing the whole PRI-E cycle at the end of the course. However, they could still listen to others' presentations and have a strong grasp of the relevant content. The students were also encouraged to participate in speech contests citywide or nationwide when they finished the PRI-E circle training. The duration of each of the two teaching methods is consistent (80 min), including before (10 min), during (60 min), and after (10 min). During the six-month rotation of learning, the frequency of the two learning methods is once a week. Moreover, there is no interaction between these two groups of students during the research period. (Fig. 3 and Fig. S1).

#### Clinical practice

Both groups learned the same clinical practice content. The major difference between the groups was the discussion. The PRI-E group engaged in a discussion before the teacher’s demonstration, and they requested preclass preparation (Figs. 3 and S1).

The details of the presentation delivery and clinical practice were controlled by a responsible staff member. The students and staff received an anonymous questionnaire before they finished the rotation.

### LBL method

The staff prepared the same learning content for the LBL group as they did with the PRI-E group. The students previewed a textbook before class and listened to the knowledge explanation provided by the staff members. The students received relevant tests when class was over. During practical skill training, the students followed the demonstration of the staff members and performed these skills by following the instructions of the staff members. Both theoretical learning and practical skill training occurred at the same frequency in the PRI-E group (Fig. 3). The students and staff received the same questionnaire after the rotation.

### Evaluation design

In September 2020, all participating students took the same entrance exam before participating in this study. Before the rotation was over, the students received the same final test and an anonymous questionnaire that asked questions about their corresponding group using a five-point Likert scale. The questionnaire contained fifteen questions concerning four different items including test-taking ability, overall ability, other evaluations, and satisfaction. The domain of Test taking ability mainly includes the ability to master basic knowledge and practical skills. The domain of Overall Ability includes critical thinking, presentation skills, problem-solving and discovery abilities, interpersonal relationships, literature search skills, team pride, and leadership. The domain of other evaluations includes engagement in learning, assistance in clinical work, and entertainment in leisure time. Moreover, the domain of satisfaction mainly includes overall satisfaction and willingness to continue using this teaching method. Each staff member is required to modify two papers from the PRI-E group and two papers from the LBL group, and during the modification process, the staff is unaware of the owners of these papers. Then they provided Likert ratings to both groups. The staff questionnaire contained ten questions concerning the students’ ability development, loads and bonds, and willingness to use in the future. Staff's questionnaire includes three domains. The domain of Ability development includes promoting critical thinking among students, helping them master basic knowledge, improving their presentation abilities, enhancing their ability to identify and solve problems, and cultivating their self-learning abilities. The domain of loads and bonds mainly includes reducing teaching burden, enhancing interaction between teachers and students, and overall satisfaction. The domain of Willingness to Use mainly includes the willingness to use two teaching methods and the willingness to promote them. Each question had five options: strongly disagree (1 point), disagree (2 points), neither agree nor disagree (3 points), agree (4 points), and strongly agree (5 points) (Tables S3 and S4).

The SWOT analysis consisted of four aspects—strengths, weaknesses, opportunities, and threats—used to evaluate both the internal and external factors that affect enterprise decision making; this is now widely used in the medical field^18–21^. A SWOT analysis was performed with the same period questionnaire survey and implementation process.

### Statistical analysis

Analysis was conducted with SPSS Statistics 26.0 (IBM Corp., Armonk, NY, USA). The Cronbach's α test was performed to evaluate the internal consistency and reliability of questionnaires. Based on the normality test (Shapiro–Wilk test) results, independent-sample t test or Mann–Whitney U test was use. Data were shown as means ± standard deviation if the variable had a normal distribution or median (lower quartile–upper quartile) in abnormal distribution. chi-square test was used to analyze unordered categorical variables. All differences between groups were observed at a statistical threshold of *p* < 0.05.

### Ethical approval

This study was conducted in accordance with the Declaration of Helsinki and was approved by the Ethics Committee of the School and Hospital of Stomatology, Wuhan University (protocol code 2022B11). All participants provided informed consent to participate in this study, and their personal information was well protected.

### Informed consent

The informed consent was obtained from all subjects for publication of identifying information/images in an online open-access publication and to participate in this study.

## Results

### Participant characteristic

Forty participants took Entrance test before evenly separated into two groups; no significant difference was found in grades. (Table 1, *P* = 0.435) According to independent-sample and chi-square test result, no difference was found either in age and gender. (Table 1).

**Table 1:** Comparison of group characteristics.

	PRI-E group	LBL group	χ^2^	t value	*p* value
Age/year (Mean ± SD)	26.25 ± 2.71	25.50 ± 2.46	/	0.916	0.365
Gender			0.100	/	0.752
Male	9	10	/	/	/
Female	11	10	/	/	/
Test Score					
Pre-test (Entrance examination)	77.90 ± 5.81	76.25 ± 7.31	/	0.790	0.435
Post-test (final examination)	81.85 ± 3.34	82.00 ± 4.42	/	− 0.121	0.904
t value	− 2.634	− 3.004	/	/	/
*p* value	0.013	0.005	/	/	/

### Final test grade

After all the courses during the rotation, PRI-E group reached 81.85 ± 3.34 while LBL group was 82.00 ± 4.42 in the final test. (*p* = 0.904) The final scores of both groups were significantly higher than the entrance scores. (Table 1).

### Student questionnaire result

The Cronbach's α of the student’s questionnaire was 0.911. The students in the PRI-E group thought that this combined learning method was superior to the traditional LBL method in terms of increasing the students’ overall abilities, such as their critical thinking, presentation skills, interpersonal relationships, and leadership according to feedback. Additionally, the PRI-E group had a higher satisfaction rate and participation than the traditional LBL learning group. However, PRI-E consumed more spare time of the students than LBL, and some students even spent two extra hours a day during the rotation, which may have led to a decrease in satisfaction. Both learning methods allowed the students to provide feedback on the clinical work in time according to the questionnaire (Table 2).

**Table 2:** Comparison of student questionnaire results.

Questionnaire Item	PRI-E group	LBL group	Mann–Whitney U	Z value	*p* value
1. test-taking ability					
This learning mode helps me master the basic knowledge of the subject	4(3–5)	4(3–4)	191.500	− 0.241	0.820
This learning mode helps me master the practical skills of the subject	4(4–4.75)	3(2–4)	104.500	− 2.700	0.009
2. Ability development					
This learning mode promotes my critical thinking	5(4–5)	3(2.25–4)	41.000	− 4.504	< 0.001
This learning mode promotes my presentation skills	5(4.25 ~ 5)	3(2 ~ 4)	40.000	− 4.572	< 0.001
This learning mode promotes students' ability to discover and deal problems	5(4.25–5)	3.5(3–4)	55.000	− 4.189	< 0.001
This learning mode improves my interpersonal skills	5(4–5)	3(3–4)	43.000	− 4.434	< 0.001
This learning mode improves my literature searching ability	4.5(4–5)	3(2–4)	53.000	− 4.131	< 0.001
This learning mode improves my team-work spirit	4(4–5)	4(3–4)	97.500	− 2.967	0.005
This learning mode promotes my leadership	4(4–5)	3(3–4)	50.000	− 4.273	< 0.001
3. Other evaluations					
This learning mode improves my participation in learning	5(4–5)	3.5(3–4)	65.000	− 3.897	< 0.001
The content of this learning mode can be well fed back into clinical work	5(4–5)	5(4–5)	164.000	− 1.164	0.341
This learning mode consumes my spare time	3(3–4)	2(2–3)	117.000	− 2.331	0.024
4. Satisfaction and willingness to use					
Overall satisfaction of the learning mode	5(4–5)	4(3–4.75)	110.000	− 2.595	0.014
I am willing to continue to use this mode in future learning activities	5(5–5)	3(3–4)	39.000	− 4.753	< 0.001
I am willing to extend this learning mode to other subjects	5(4.25–5)	4(3–4.75)	82.500	− 3.472	0.001

### Staff questionnaire result

Ten staff members participated in the teaching activities of both groups, and they evaluated the teaching details of both groups at the end of the study. The Cronbach's α of the staff’s questionnaire was 0.953. The PRI-E group received a higher grade in the ability development of the students, which concerned skills such as critical thinking, problem solving, and independent learning. The PRI-E mode enhanced the bonds between the students and teachers due to the interactive discussion form. However, we found no significant difference in teaching load between the two groups, which indicated that PRI-E was still a teacher-led method that required elaborate design and guidance. All the staff members showed interest in and enthusiasm about PRI-E for future use. (Table 3).

**Table 3:** Comparison of staff questionnaire results.

Questionnaire Item	PRI-E group(N = 10)	LBL group(N = 10)	Mann–Whitney U	Z value	*p* value
1.Ability development					
This learning mode promotes students' critical thinking	5(4–5)	3(3–3)	6.500	− 3.513	< 0.001
This learning mode helps students master the basic knowledge of the subject	4(3–5)	3(2–3)	10.500	− 3.215	0.001
This learning mode promotes students' presentation skills	5(5–5)	3(2–3)	0.500	− 3.990	< 0.001
This learning mode promotes students' ability to discover and deal problems	5(4–5)	3(2.75–3.25)	3.000	− 3.713	< 0.001
This learning mode promotes students' independent learning	5(5–5)	3(3–3)	0.000	− 4.192	< 0.001
2.Loads and bonds					
This mode of learning makes teaching easier	4(3–5)	3.5(3–4)	38.500	− 0.923	0.356
This mode of learning enhances the bond between teachers and students	5(4.75–5)	3(2.75–3)	1.000	− 3.924	< 0.001
Overall satisfaction of the learning mode	5(4–5)	3(2.75–3.25)	3.000	− 3.713	< 0.001
3.Willingness to use (grade ≥ 4)					
I am willing to continue to use this model in future teaching activities	10(100%)	0 (0%)	/	/	< 0.001
I am willing to extend this learning mode to other subjects	10(100%)	0 (0%)	/	/	< 0.001

### SWOT analysis

**Figure 4:**
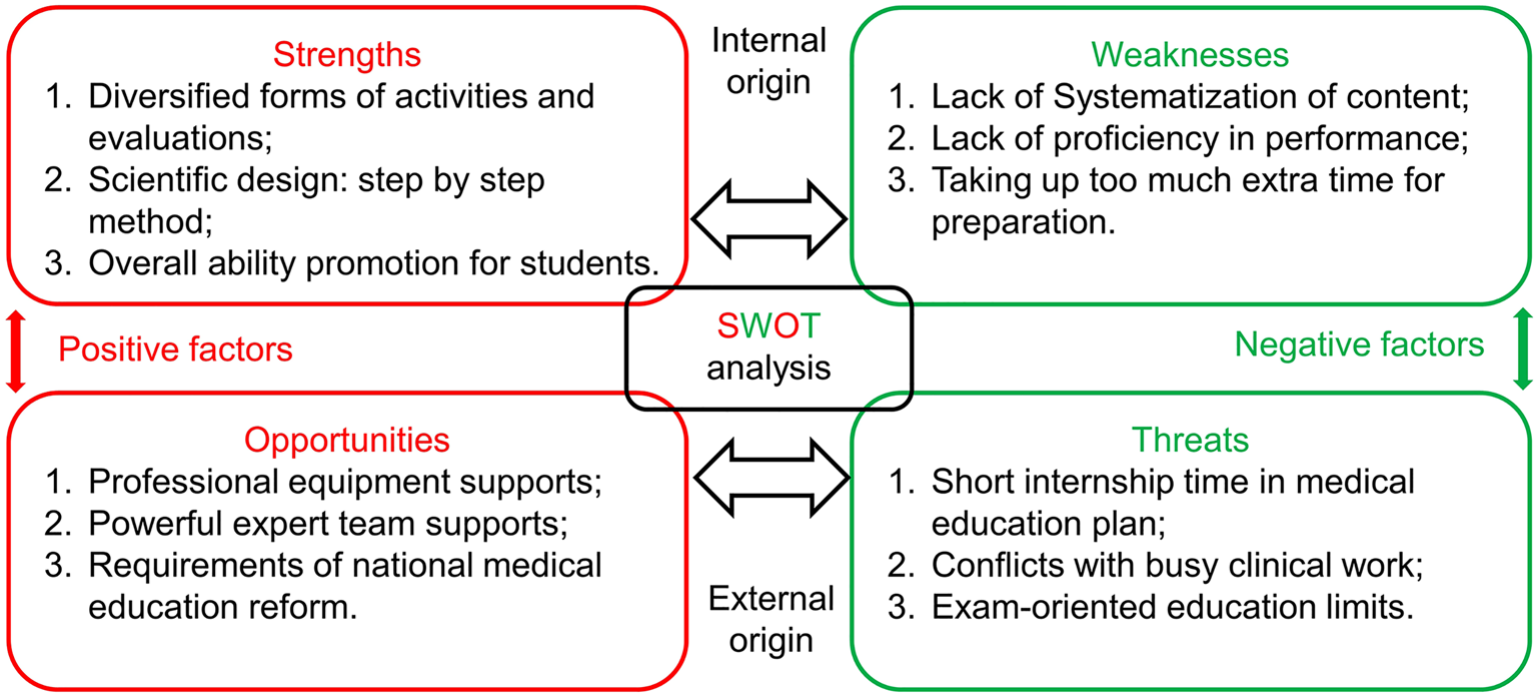
SWOT Analysis result. The advantages, opportunities, weaknesses and threats of PRI-E teaching method are analyzed in detail through SWOT method.

The overall assessment is presented in the form of a SWOT analysis in Table S5 and Fig. 4.

#### Strengths

Benefiting from the combination of various teaching modes, students can greatly strengthen their comprehensive abilities in the process. PRI-E adopts a step-by-step approach to guide students through orderly progress. This individualized teaching mode allows students to clearly observe their own path of progress and grasp the essence of all the different learning modes (PBL, CBL, and EBL).

#### Weaknesses

The lack of systematic teaching content leads to low efficiency in a single class. The teaching effect may be affected by the lack of relevant teaching experience of staffs in the department. Taking too much extra time may cause students’ dissatisfaction with the learning and bring inconvenience in daily life.

#### Opportunities

Department of Oral & Maxillofacial Head Neck Oncology, School & Hospital of Stomatology, Wuhan University has good equipment support, which enable students to get special training like microscope suture, hand-held doppler ultrasound detection.

#### Threats

Short internship time and busy clinical work can adversely affect learning outcomes. PRI-E took up more extra time and it is bound to cause students' anxiety and fluctuations in grades in the context of exam-oriented education.

## Discussion

At present, the LBL teaching method is still the main way to conduct medical education in China, but it is not conducive to the overall development of students^22^. LBL method is not conducive to the cultivation of good clinical thinking of students, nor does it help students' presentation ability^23^. The PBL method can be used to effectively increase students' awareness of active learning. However, it seems to increase the burden on students and still does not help their overall development^24^. So it still cannot be used to meet the teaching requirements of oral and maxillofacial oncology^22–26^. In order to solve the problems of the existing learning methods and make the teaching methods better adapt to the teaching needs of oral and maxillofacial oncology, based on a variety of previous learning methods, we designed the PRI-E learning method.

We conducted a questionnaire survey on all the participants. The results showed that the PRI-E learning method was more popular among the participants. Compared with the traditional LBL method, the PRI-E method showed higher teaching satisfaction and was considered to be more conducive to future career development by the participants. During the implementation of the PRI-E method, this step-by-step method enabled students to make a comprehensive and objective judgment on the lecturer's teaching content. In addition, students can also exercise their interpersonal and communication skills with patients during PRI-E learning, which is not possible in traditional LBL learning. In addition, although some students did not complete the whole PRI-E process, they claimed that their learning was also promoted through other people's presentations, and they gained a plethora of knowledge. In addition, we also allowed undergraduate students interested in oral and maxillofacial oncology to participate in the audit. In future work, we will also promote this teaching mode to undergraduate teaching so as to expand the audience of this learning mode.

By comparing the course examination results after learning with the traditional LBL method, we found that the PRI-E method did not cause a decline in the students' examination scores. These results showed that the PRI-E method has more benefits on the basis of not affecting academic performance. These benefits include a more effective enhancement of the students' learning interest and an increased promotion of students' career development. The PRI-E method requires more of the students’ time than the LBL method, but this is not rejected by students. This is because students are more willing to devote their time to the courses as a result of the increase in their interest. Therefore, the PRI-E method can promote students' future development without affecting their test results. Moreover, the PRI-E method can also stimulate students' interest in learning and promote students to invest more time in the study of oral and maxillofacial oncology.

The SWOT analysis was conducted according to the feedback and discussion of the staff team. In terms of internal factors, PRI-E had strengths such as diversified learning forms. Although the process is supervised by teachers, the learning process is student centered. The presentations were conducted in an equal and harmonious environment. The students gained experience and confidence after completing their first presentation, which encouraged them to perform more accurately in the next round. The step-by-step approach was an individualized method that could make the PRI-E mode more proper and efficient. However, using too much time may arouse dissatisfaction. Due to the difference between the traditional learning modes, the staff also needed time to accumulate experience, which led to low efficiency at the starting stage. The topic setting was not systematic at the beginning, and the presentation content had little correlation with the topic. We optimized this flaw in the later process by organizing the topic presentation; for instance, if the topic was on cystic lesions of the mandible, all participants delivered presentations related to cystic lesions of the mandible. The PBL presentation showed the clinical manifestations and the latest classification whereas the CBL presenter shared real cases of different disease types. EBL requires assignment by staff members to summarize treatment-selective criteria for different lesions. Although medical teaching reform is advocated in China, time is still required to change the examination-oriented education model being applied. The PRI-E group participants and staff agreed that the PRI-E mode was more effective at developing doctors' comprehensive abilities, but they were still worried about failing the corresponding exam. We plan to include a certain proportion of basic knowledge review and testing in future PRI-E activities to compensate for this deficiency. With the accumulation of experience, we will gradually define the details of the activities and enrich the educational philosophy of PRI-E.

This study has some limitations. Although the PRI-E learning method showed considerable advantages, its use did not effectively increase the students' test scores. This situation also appears in the research of other scholars, which is a common problem when developing new learning methods^27,28^. We analyzed two reasons for this result: a lack of review guidance for exam-oriented content, and requiring too much extra time from students. Although the use of the PRI-E method resulted in high scores from students and teachers regarding ability development and classroom participation, it lacked examination guidance. Specific enhancements should be planned in future activities.

## Conclusion

We compared the PRI-E learning mode with the traditional LBL learning mode. PRI-E activities were guided by a clear teaching concept of Passion, Research, and Innovation. The PRI-E mode performed more strongly in terms of ability development, which included skills such as critical thinking, finding and solving problems, interpersonal communication, etc. In addition, they had a higher satisfaction rate according to the questionnaire results. However, the PRI-E mode still needs to be perfected in terms of enhancing test scores and reducing learning burden.

### Supplementary Information

Supplementary Information.

## Data Availability

The datasets used and/or analyzed during the current study are available from the corresponding author upon reasonable request.
