# Supplementary material for: Application of PRI-E–a combined learning method in oral and maxillofacial oncology education
Source: Sci Rep. 2024 Apr 7;14:8127. doi: 10.1038/s41598-024-58878-y (PMC10999407; doi:10.1038/s41598-024-58878-y)
Supplement: Supplementary file 1 — Supplementary Information. [file 41598_2024_58878_MOESM1_ESM.docx]

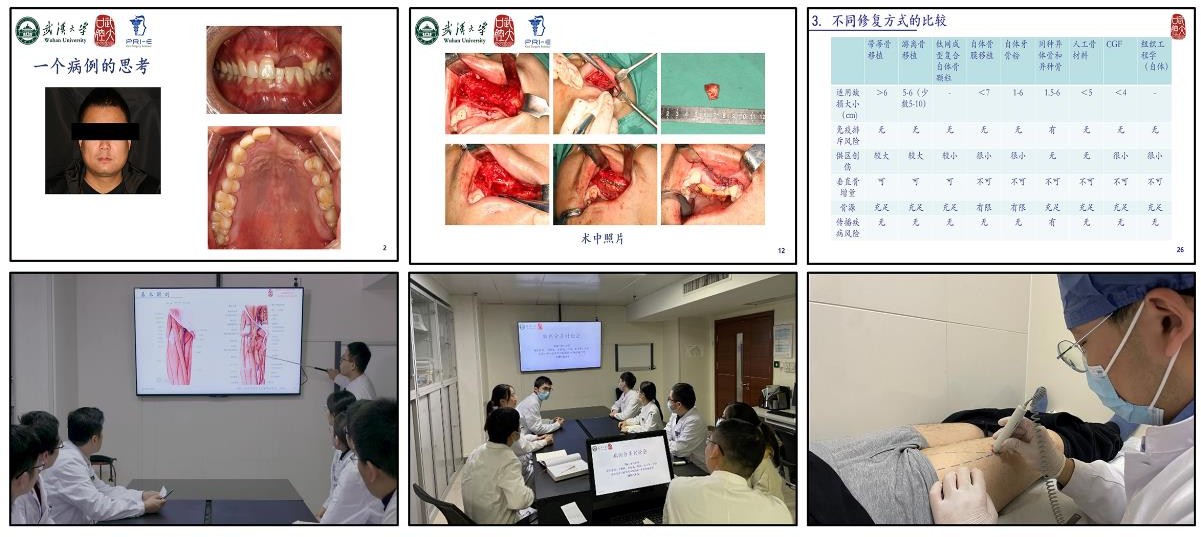


**Fig.S1.** PRI-E activity images. This figure shows the teaching scenes and courseware of PRI-E.

**Table S1. Pri-Examination (Entrance Examination)**

1.Which of the following clinical manifestations is unique to sebaceous gland cysts

A. Commonly seen on the face

B. Slow occurrence

C. The cyst wall adheres to the skin, and there may be a pigment spot in the center

D. Soft texture, no tenderness Clear boundaries, movable

2.The main difference between dermoid cysts and epidermoid cysts is

A. There is no skin accessory structure in the cyst wall of dermoid cysts

B. There is no skin accessory structure in the cyst wall of epidermoid cysts

C. There are skin adnexal structures in the cyst wall of epidermoid cysts

D. Dermatoid cysts do not contain keratinization Epidermal cyst without keratinization

3. The presence of skin attachments in the cyst cavity may be

A. Sebaceous gland cyst

B. Primordial cyst

C. Dermoid cyst

D. Epidermal cyst

4. Dermatoid cysts are caused by

A. Formation of tumor cell proliferation and division

B. The development of epithelial cells left in tissues during embryonic development

C. During embryonic development, endodermal cells are left to develop and form

D. Formation of sebaceous gland cyst expansion

5. Which of the following is a unique feature of dermatoid or epidermoid cysts

A. Slow growth

B. More common in children and young people

C. Palpation is tough and elastic, resembling a dough

D. Clear boundaries

6. The most common location for thyroglossal duct cysts is in the midline of the neck

A. Root of tongue

B. Lower part of hyoid bone

C. Upper and lower parts of the hyoid bone

D. Upper part of hyoid bone

7. Regarding thyroglossal duct cyst, which one is incorrect

A. Occurred in any part of the anterior midline of the neck

B. Cyst connected to lingual foramen

C. Cysts move up and down with swallowing

D. Surgical treatment should include partial removal of thyroid cartilage

8.To prevent postoperative recurrence of thyroglossal cyst or fistula, surgery should

A. Complete removal of cyst

B. Tracing fistula to hyoid surface ligation

C. The cyst peeled off completely on the surface of the hyoid bone

D. Remove the cyst and the middle part of the hyoid bone, and properly handle the part above the hyoid bone

9. The second parotid cleft cyst is often located in

A. Near the mastoid process

B. Near the mandibular angle

C. Cervical anterior midline

D. Hyoid level, near the anterior edge of the upper one-third of the sternocleidomastoid muscle

10. Which of the following is not a manifestation of multiple basal cell nevus syndrome

A. Multiple jawbone odontogenic keratocysts

B. Skin basal cell nevus

C Bifurcated rib

D. Calcification of cerebral falx

11. The most common source of branchial cleft cysts in clinical practice is from

A. First gill fissure

B Second gill fissure

C. Third gill fissure

D. Fourth gill fissure

12. What belongs to odontogenic cysts

A. Bulbar maxillary cyst

B Root cyst

C. Nasolabial cyst

D. Maxillary median cyst

13. Odontogenic keratocysts belong to

A. Developmental jawbone cyst

B. Pseudocyst of jawbone

C. Odontogenic jawbone cyst

D. Embryonic jawbone cyst

14. Cysts caused by inflammation in the oral and maxillofacial regions are mainly

A. Root cyst

B. Mucinous cyst

C. Sublingual gland cyst

D. Thyroglossal duct cyst

E. Sebaceous gland cyst

15.Cysts that often contain keratinization may be

A. Branchial cleft cyst

B. Thyroglossal duct cyst

C. Facial fissure cyst

D. Dermatoid and epidermoid cysts

**Table S2. Post-examination (final exam)**

1. The place with the highest incidence rate of oral cancer is

A. Tongue B. Cheeks C. Hard palate D. Lips

2. Tumors that are sensitive to radiotherapy include

A. Squamous cell carcinoma

B. Malignant melanoma

C. Osteosarcoma

D. Chondrosarcoma

3. Tumors that can move with swallowing and tongue extension include

A. Thyroid bone duct cyst

B. Branchial cleft cyst

C. Ameloblastoma

D. Tongue cancer

4. Oral cancer can easily cause restricted oral opening

A. Tongue cancer

B. Oral cancer

C. Maxillary sinus cancer

D. Cancer of the bottom of the oral cavity

5. What is the most common malignant tumor in the oral and maxillofacial regions

A. Squamous cell carcinoma

B. Malignant melanoma

C. Osteosarcoma

D. Chondrosarcoma

6. Non odontogenic cysts

A. Spherical maxillary cyst B. Primitive cyst C. Topical cyst D. branchial cleft cyst

7. Which part of adenoid cystic carcinoma is most prone to metastasis

A. Lung B. Liver C. Kidney D. Bone

8. What is the most suitable treatment method for osteosarcoma

A. Surgical treatment B. Radiotherapy C. Cryotherapy D. Chemotherapy

9. Which of the following vascular diseases can cause venous stones

A. Wine spot capillary hemangioma

B. Yangmei like capillary hemangioma

C. Vascular nevus

D. Cavernous hemangioma

10. The main types of oral and maxillofacial cysts caused by inflammation include

A. Top cyst B. Mucous cyst C. Sublingual cyst D. Primitive cyst

11. Parotid gland masses usually do not undergo preoperative pathological examination, but the main reason for intraoperative frozen examination is

A. If there is a facial nerve, sampling is not easy

B. The patient will avoid the pain of two surgeries

C. Increase the chance of blade infection

D. Increasing the difficulty of facial nerve anatomy does not comply with the principles of tumor treatment

12. Which of the following is incorrect

A. Malignant tumors come from different tissues and have different treatment methods.

B. According to the extent of tumor invasion, the International Association for Anti- Cancer has developed a TNM classification.

C. T represents primary tumor.

D. N represents tumor size

13. Which gland is the most common site of mucoepidermoid carcinoma

A. Parotid gland B. submandibular gland C. sublingual gland D. labial gland

14. Tumors with obvious wave motion include

A. Squamous cell carcinoma B. Adenoid cystic carcinoma C. Giant cell tumor of bone

D. Gingival tumor

15. The main side effects of tumor chemotherapy are:

A. Gastrointestinal reactions

B. Hair loss

C. Liver injury

D. Bone marrow transplantation

# Table S3. Student Questionnaire

1. **Your group is**

1 group 2 groups

# What is your gender?

1. Male 2. Female

# What is your age?

1. **How does this model help you master basic knowledge?**

5. Very significant 4. Significant 3. Average 2. Not significant 1. Very insignificant

# How does this mode help to master operation skills?

5. Very significant 4. Significant 3. Average 2. Not significant 1. Very insignificant

# Can the thematic learning conducted be well fed back to the clinical work?

5. Very significant 4. Significant 3. Average 2. Not significant 1. Very insignificant

# Does this mode consume too much spare time for you?

5. Very significant 4. Significant 3. Average 2. Not significant 1. Very insignificant

# Average time spent per day?

1. ＜15min 2. 15-30min 3. 30-60min 4.＞60min

# How does this model improve your critical thinking?

5. Very significant 4. Significant 3. Average 2. Not significant 1. Very insignificant

# How does this model improve your expression ability (speech, PPT production)?

5. Very significant 4. Significant 3. Average 2. Not significant 1. Very insignificant

# How does this model improve your ability to find and solve problems?

5. Very significant 4. Significant 3. Average 2. Not significant 1. Very insignificant

# How does this model improve your interpersonal skills?

5. Very significant 4. Significant 3. Average 2. Not significant 1. Very insignificant

# How does this model improve your literature retrieval ability?

5. Very significant 4. Significant 3. Average 2. Not significant 1. Very insignificant

# How does this model improve your teamwork ability?

5. Very significant 4. Significant 3. Average 2. Not significant 1. Very insignificant

# How does this model improve your leadership?

5. Very significant 4. Significant 3. Average 2. Not significant 1. Very insignificant

# How much participation do you have in this model?

5. Very significant 4. Significant 3. Average 2. Not significant 1. Very insignificant

# What is your overall satisfaction with this model?

5. Very significant 4. Significant 3. Average 2. Not significant 1. Very insignificant

# Are you willing to use this method in other disciplines?

5. Very significant 4. Significant 3. Average 2. Not significant 1. Very insignificant

# Are you willing to lead others to implement this learning mode in their future learning life?

5. Very significant 4. Significant 3. Average 2. Not significant 1. Very insignificant

# What do you think is the disadvantage of this model?

**Table S4. Tutor Questionnaire**

# Do you think PRIE can improve students' critical thinking?

5. Very significant 4. Significant 3. Average 2. Not significant 1. Very insignificant

# Do you think the traditional teaching mode can improve students' critical thinking?

5. Very significant 4. Significant 3. Average 2. Not significant 1. Very insignificant

# Do you think PRIE can improve students' mastery of basic knowledge?

5. Very significant 4. Significant 3. Average 2. Not significant 1. Very insignificant

# Do you think the traditional mode can improve students' mastery of basic knowledge?

5. Very significant 4. Significant 3. Average 2. Not significant 1. Very insignificant

# Do you think PRIE can improve students' expression ability (speech, PPT presentation)?

5. Very significant 4. Significant 3. Average 2. Not significant 1. Very insignificant

# Do you think the traditional model can improve students' expression ability (speech, PPT presentation)?

5. Very significant 4. Significant 3. Average 2. Not significant 1. Very insignificant

# Do you think PRIE can improve students' ability to find and deal with problems?

5. Very significant 4. Significant 3. Average 2. Not significant 1. Very insignificant

# Do you think the traditional model can improve students' ability to find and deal with problems?

5. Very significant 4. Significant 3. Average 2. Not significant 1. Very insignificant

# Do you think PRIE can improve students' ability to learn independently?

5. Very significant 4. Significant 3. Average 2. Not significant 1. Very insignificant

# Do you think the traditional model can improve students' ability to learn independently?

5. Very significant 4. Significant 3. Average 2. Not significant 1. Very insignificant

# Do you think PRIE makes teaching easier?

5. Very significant 4. Significant 3. Average 2. Not significant 1. Very insignificant

# Do you think the traditional mode makes teaching easier?

5. Very significant 4. Significant 3. Average 2. Not significant 1. Very insignificant

# Do you think PRIE has improved the relationship between teachers and students?

5. Very significant 4. Significant 3. Average 2. Not significant 1. Very insignificant

# Do you think the traditional model has improved the relationship between teachers and students?

5. Very significant 4. Significant 3. Average 2. Not significant 1. Very insignificant

# How satisfied are you with the PRIE teaching model?

5. Very significant 4. Significant 3. Average 2. Not significant 1. Very insignificant

# How satisfied are you with the traditional teaching model?

5. Very significant 4. Significant 3. Average 2. Not significant 1. Very insignificant

# Are you willing to continue to use the PRIE mode in the future teaching work?

5. Very significant 4. Significant 3. Average 2. Not significant 1. Very insignificant

# Do you think PRIE is worth promoting in the teaching of other disciplines?

5. Very significant 4. Significant 3. Average 2. Not significant 1. Very insignificant

# Other comments and suggestions?

**Table S5. Questionnaire for SWOT analysis**

1. What are the main problems you encounter when using this learning mode?

2. In what ways does this learning mode help improve your abilities?

3. What opportunities and challenges do you think this learning model brings?

4.What do you think is the future development direction of learning models?

5.What do you think are the main shortcomings of this learning model?

6.Do you have any other ideas to share about teaching oral and maxillofacial head and neck oncology?
